# A biodegradable ocular implant for long-term suppression of intraocular pressure

**DOI:** 10.1007/s13346-015-0240-4

**Published:** 2015-06-23

**Authors:** Xu Wen Ng, Kerh Lin Liu, Amutha Barathi Veluchamy, Nyein Chan Lwin, Tina T. Wong, Subbu S. Venkatraman

**Affiliations:** School of Materials Science and Engineering, Nanyang Technological University, Block N4.1-02-06, Nanyang Avenue, Singapore, 639798 Singapore; Singapore Eye Research Institute (SERI), Singapore National Eye Centre, Level 5, 11 Third Hospital Avenue, Singapore, 168751 Singapore

**Keywords:** Biodegradable, Polymer, Blend, Glaucoma, Sustained release

## Abstract

Timolol maleate (TM) has been used for many years for the reduction of intraocular pressure (IOP) in glaucoma patients. However, the topical mode of administration (eyedrops) is far from optimal because of the issues of low bioavailability, high drug wastage, and lack of patient compliance. Suboptimal control of the IOP leads to disease progression and eventually to blindness. Ideally, TM is delivered to the patient so that its action is both localized and sustained for 3 months or more. In this work, we developed a subconjunctival TM microfilm for sustained, long-term delivery of TM to the eyes, using the biodegradable elastomer poly(lactide-co-caprolactone) (PLC). The copolymer is biocompatible and has flexibility and mechanical characteristics suitable for a patient-acceptable implant. Controlling the release of TM for 3 months is challenging, and this work describes how, by using a combination of multilayering and blending with poly(ethylene glycol) (PEG) copolymers, we were able to develop a TM-incorporated biodegradable film that can deliver TM at a therapeutic dose for 90 days in vitro. The data was further confirmed in a diseased primate model, with sustained IOP-lowering effects for 5 months with a single implant, with acceptable biocompatibility and partial degradation.

## Introduction

Glaucoma is a progressive optic neuropathy that is characterized by characteristic changes to the optic nerve and visual field loss. Glaucoma is an age-related eye disease and affects 60.5 million people worldwide [1]. According to the World Health Organization (WHO) statistics, glaucoma is the second leading cause of blindness in the world [2]. Based on the projected expansion of the aging population, it is estimated that by 2020, glaucoma would affect about 80 million people, leaving close to 11 million bilaterally blind [1]. This will inevitably result in the loss or productivity, as well as in cost for the healthcare system as a whole.

Although glaucomatous syndromes could be categorized as normal-tension glaucoma (NTG) and high-tension glaucoma (HTG), the elevation of intraocular pressure (IOP) is the major modifiable risk factor of glaucoma and, if left untreated, results in disease progression in 70 % of all glaucoma cases [3]. According to the Early Manifest Glaucoma Treatment trial, for every 1-mmHg reduction in IOP, the risk of glaucoma progression is decreased by 10 % [4]. One of the most widely used medical treatments is timolol maleate (TM) eyedrops. Timolol, which was approved by the FDA in 1979 [5], is a beta-adrenergic receptor antagonist that reduces the IOP by decreasing the production of aqueous humor [5]. Beyond its excellent IOP-lowering efficacy, the effectiveness of the treatment outcome is variable and highly dependent on patient compliance. One recent clinical survey observed that nearly 80 % of the patients who are diagnosed with glaucoma discontinued the treatment via eyedrops after 1 year, and this number increased to ∼90 % after 3 years [6]. Moreover, this mode of administration is suboptimal because of low bioavailability of the drug, caused by rapid elimination in the pre-corneal area via lacrimation, tear drainage, and turnover.

Sustained drug delivery offers the best alternative to overcome low drug bioavailability and drug fluctuation issues and helps eliminate the issue of patient compliance. Beyond patient convenience and compliance, greater therapeutic efficacy could be achieved. Efforts to develop such TM delivery systems have been ongoing, and a number of different systems have emerged. They include fornex inserts [7], hydrogels [8], contact lenses [9–11], nano-fiber [12], and microspheres [13]. However, these systems have not made it to the clinic primarily because they lack sustained release: the typical release period is a few hours to days [7–12] and does not extend to a month or more. Recently, Bertram et al. reported a blend microsphere system that achieved long-term release of TM [13] via subconjunctival injection; however, there was little discussion of the daily targeted dose and whether it was achieved. Moreover, due to the fairly large particle size and relatively low drug loading in the microspheres, it is likely that the required volume per injection is substantial. Hence, to date, there is no viable TM delivery system that can be readily translated for clinical use.

Therefore, the goal of this work is to develop a timolol delivery system that allows placement in the subconjunctiva while delivering timolol in a consistent and controlled manner. Timolol is typically administrated via eyedrops twice a day as a 0.25 % formulation, which amounts to a daily dose of about 250 μg. The bioavailability of timolol delivered through eyedrops is only about 1–2 %, indicating that the effective daily dosage required is 2.5–5 μg/day. In this work, we evaluated the release of timolol maleate from microfilms composed of poly(lactide-co-caprolactone) (PLC)- and poly(ethylene glycol) (PEG)-based copolymers, with the focus of achieving the targeted daily dosage of ∼2.5 μg for 3 months. The subconjunctival implantation of this microfilm is done in a minimally invasive mode that will be patient acceptable. We believe this work is by far the longest sustained delivery of timolol reported to date, achieving the targeted daily dose. To verify the in vitro to in vivo translatability, the implant was inserted into the subconjunctival space of primate eyes and was shown to be safe and efficacious for up to 5 months. The results suggest strongly that this system offers a paradigm shift in treating glaucoma.

## Materials and methods

### Materials

Granular 70:30 poly(lactide)/poly(ε-caprolactone) copolymer (PLC, Purac), poly(ε-caprolactone)/poly(ethylene glycol) (PCL-PEG, Advanced Polymer Materials Inc.), timolol maleate salt (TM, Sigma), timolol base (TB, Nivon Pharma), phosphate buffer saline tablets (pH 7.4, PBS, Sigma), and ammonium acetate (Sigma-Aldrich) were used as received. Dichloromethane (DCM, Tedia Chemical Company Inc.), acetonitrile (ACN, Tedia Chemical Company Inc.), methanol (MeOH, Tedia Chemical Company Inc.), and triethylamine (TEA, Sigma-Aldrich) used were of HPLC grade.

### Film preparation

#### Single layer

TM or TB was first dispersed/dissolved in DCM followed by the addition of polymeric pellets at 1 g polymer to 5 mL DCM ratio. The mixture was stirred overnight to obtain a homogenous TM/TB-loaded polymer solution. The solutions were cast onto a glass plate and dried under ambient conditions overnight before drying in a vacuum oven at 37 °C for 1 week. The preparations were conducted in the absence of light due to the light sensitivity of TM and TB. All the formulations are listed in Table 1.

**Table 1:** Compositions of the single-layer films

Sample name	Polymer ratio (%)		Drug loading (wt% of total film)		Thickness (μm)
	PLC	PCL (10 k)-PEG (5 k)	TM	TB	
PLC	–	–	–	–	40
PLC 1% TB	100	–	–	1	40
PLC 5% TB	100	–	–	5	40
PLC 1% TM	100	–	1	–	40
PLC 5% TM	100	–	5	–	40
90:10 PLC/PCL-PEG 1% TM	90	10	1	–	40
80:20 PLC/PCL-PEG 1% TM	80	20	1	–	40
90:10 PLC/PCL-PEG 5% TM	90	10	5	–	40
80:20 PLC/PCL-PEG 5% TM	80	20	5	–	40

#### Multilayer

TM was first dispersed in DCM followed by the addition of polymeric pellets at a 1 g polymer to 5 mL DCM ratio. The mixture was stirred overnight to obtain a homogenous TM-loaded polymer solution. A blank polymer solution was prepared in a similar manner. The blank polymer solution was first cast onto a glass plate, followed by the TM-loaded polymer solution on top of the first blank polymer layer and a final blank polymer solution on top of the TM-loaded layer at 3-min intervals. The dry thickness of the film is well correlated to the wet thickness of the film as long as factors such as drying environment, casting rate, casting platform, and polymer solution concentration are kept constant. The wet thickness of the film was adjusted such that the dry thickness is about 15 μm for the two blank layers and about 10 μm for the TM-loaded layer. The multilayered films were dried under ambient conditions overnight before drying in a vacuum oven at 37 °C for 1 week. The preparations were conducted in the absence of light due to the light sensitivity of TM. Finally, the thickness of the film was measured using the Elcometer 456. All the formulations are listed in Table 2.

**Table 2:** Compositions of the multilayered films

Sample name	Layer	Schematic	Drug loading (wt% of total film)	Dry thickness (μm)
Sandwich PCL (10 k)-PEG (5 k) 5% TM	1	PLC	–	15
	2	5 % timolol maleate in 80:20 PLC/PCL (10 k)-PEG (5 k)	5	10
	3	PLC	–	15
Sandwich PCL (10 k)-PEG (5 k) 20 % TM	1	PLC	–	15
	2	20 % timolol in 80:20 PLC/PCL (10 k)-PEG (5 k)	20	10
	3	PLC	–	15
Sandwich PCL (10 k)-PEG (1 k) 20 % TM	1	PLC	–	15
	2	20 % timolol in 80:20 PLC/PCL (10 k)-PEG (1 k)	20	10
	3	PLC	–	15

### In vitro release studies

In vitro release studies were conducted by incubating the films of 10 × 10 mm at 37 °C in glass bottles, each containing 3 mL of buffer solution (pH 7.4). At each sample retrieval time point, the release medium was completely removed and replaced with fresh buffer to maintain sink condition. The TM and TB concentrations in the release medium were analyzed using reversed-phase higher performance liquid chromatography (RP-HPLC). *TM analysis:* An Agilent Zorbax Eclipse XDB C-18 column (4.6 × 250 mm, 5 μm) was connected with a Dionex 3000-RS equipped with a diode array detector (DAD). Mobile phase 25:75 volume ratio of ACN/20 mM ammonium acetate was used where the pH of the mobile phase was adjusted to pH 5.2 with acetic acid. Other operating conditions were as follows: flow rate = 1 mL/min, sample volume = 10 μL, column temperature = 30 °C, and detector = 294 nm. *TB analysis:* An Agilent Zorbax Extend C-18 column (4.6 × 250 mm, 5 μm) was connected with a Dionex 3000-RS. Mobile phase 30:70 volume ratio of 20 mM TEA/methanol was used. Other operating conditions were as follows: flow rate = 1 mL/min, sample volume = 10 μL, column temperature = 30 °C, and detector = 299 nm. All samples were prepared and tested in triplicates while the data is presented as mean ± standard deviation of the mean.

### Polymer degradation

The degradation of polymers was characterized in terms of the changes in mass and molecular weight. Films of 10 × 10 mm dimensions were immersed in buffer solution (pH 7.4) and incubated at 37 °C. The buffer was refreshed weekly and at every predetermined time point; some films were removed from the buffer, rinsed with deionized water, and dried in a 37 °C vacuum oven for at least 7 days. The mass of the dried samples was measured followed by molecular weight measurement using a gel permeation chromatography (GPC, Agilent 1100) equipped with refractive index detector (RID). Fifty microliters of the degraded polymer samples in chloroform was injected through an Agilent PLGel MIXED-C column (300 × 7.5 mm) under a constant chloroform flow rate of 1 mL/min at 35 °C. The molecular weight of the samples was obtained relative to a calibration curve from polystyrene standards (Mw between 165 and 5000 g/mol).

### Film sterilization for in vivo studies

All samples were sterilized using ethylene oxide at 37 °C for 24 h prior to insertion into monkeys.

### Animal study: monkey model of glaucoma

Experimentation on non-human primates (*Macaca fascicularis*) was performed in accordance with the statement for the use of animals in ophthalmic and vision research approved by the Association for Research in Vision and Ophthalmology. The guidelines of the Animal Ethics Committee of the SingHealth Singapore Association for Assessment and Accreditation of Laboratory Animal Care (International-accredited) were also satisfied. Non-human primates were anesthetized by intramuscular injection of ketamine (20 mg/kg body weight) and acepromazine maleate (0.25 mg/kg body weight). Their airway, respiration, and pulse were monitored during all procedures. One to two drops of 1 % xylocaine were used as topical anesthesia to reduce possible discomfort to the animals involved during the procedure.

Nine non-human primates were used in this study and were divided into two groups: group 1, high-intraocular-pressure (hypertensive) monkeys (*n* = 6) received a single subconjunctival implantation of 4 mm × 6 mm timolol microfilm into both eyes, and group 2, hypertensive monkeys (*n* = 3) received twice-daily timolol eyedrops into both eyes for 28 days.

### Subconjunctival implantation of timolol microfilms in non-human primates

This procedure was performed under aseptic conditions in a surgical theater equipped for non-human primates. The group 1 non-human primates received a subconjunctival implant with timolol-loaded microfilm. In brief, a limited conjunctival dissection was performed and then a microfilm was inserted; it was sutured 2 mm posterior to the limbus and secured with two 10/0 nylon sutures. Topical tobramycin ointment 1 % was administered to the operated eye daily for 5 days.

### Intraocular pressure monitoring and clinical examination

For IOP measurements, the non-human monkeys were lightly anesthetized with ketamine at 5 mg/kg body weight. The topical anesthesia was applied as mentioned above. IOP was measured via tonometer (Tono-Pen® XL, Reichert Technologies, Depew, NY) at 2–4 p.m. weekly for 1 month, and biweekly for the second month and every month for another 2 months. The procedure took about 5 min for each monkey. Once daily baseline IOP measurements to both eyes of all animals for three consecutive days were recorded prior to the commencement of the treatment at 2–4 p.m. Six to eight IOP measurements were taken to ensure an average IOP measurement is attained each time. The treatment was commenced on day 4.

### Clinical examinations

Non-human primates were anesthetized, and visual inspection of all eyes after injections, or topical administration, was done every day for signs of conjunctival irritation, inflammation, or infection at the injection site. Slit lamp microscopic examination of the exterior, anterior chamber, and posterior chamber of the eyes was performed before the injections and weekly thereafter. The monkeys were also monitored for any gross changes such as eye discharge, squinting, or abnormal behavior suggesting pain or severe discomfort.

### Mathematical model

The power law model [14, 15] is a semi-empirical equation used to describe drug release from polymeric systems. The model describes a biphasic release, a fast initial release due to desorption followed by a diffusion-controlled release as can be seen from the summation of the two factors in the equation.

$$ \frac{M_t}{M_{\infty }}=b+a{t}^n $$

where *b* represents the initial (sometimes called the “burst” release) by desorption, $a$ represents a constant that is governed by structure and geometric characteristics, and $n$ is an exponent that is related to the mechanistic details of the release: at the Fickian limit, the value of *n* is 0.5 for thin films. For this work, single-layered formulations were fitted to the equation. Since the formulations are monolithic matrices, *n* is expected to be close to 0.5 in this case.

### Statistical analysis

Significance was set at the 0.05 level. SPSS version 19 (IBM SPSS Statistics, Chicago, USA) was used for all analyses.

## Results and discussion

Currently, topical application of timolol remains the most common treatment option for reducing IOP in glaucoma and ocular hypertension patients. Topical administration of medical eyedrops is suboptimal and involves high wastage of the drug, and cannot be sustained for more than a few hours. Therefore, for chronic conditions, poor patient compliance is a major challenge. Although there is a lot of dissatisfaction with the eyedrop timolol treatment, there is no breakthrough in treatment options due to the stringent dose requirements over long periods; where sustained delivery has been reported in in vitro studies, there is no convincing in vivo data to show enhanced duration of action. Hence, in this work, we report on the development of a “soft” microfilm that can deliver the dose of 2.5 μg of timolol daily for several days. The elastomer PLC was chosen as the main microfilm matrix because it is biocompatible and our laboratory has demonstrated its use in ocular biomedical devices [16–18]. Moreover, the eventual degradation of this copolymer into harmless by-products would eradicate the need for removal surgery.

### Strategies to control burst release

In order to achieve sustained delivery for 3 months, the following strategies were pursued:

- Use of the base form of TM to enhance lipophilicity and thus sustain its release longer
- Blending of PLC with PEG copolymers to reduce burst effects and to sustain release, especially for high drug loading
- Use of a sandwich film, i.e., three-layered film with the drug-incorporated layer in the middle to minimize burst and sustain release

#### Effects of drug form

Timolol drugs exist in two forms—an esterified form known as timolol maleate, TM, and a “base” form known as timolol, TB. In the usual case of amine-based drugs, the base form is considered more lipophilic than the salt form; consequently, the burst effect of the drug (from lipophilic matrices such as PLC) is less when the drug is in the base form, as it is more soluble.

**Fig. 1:**
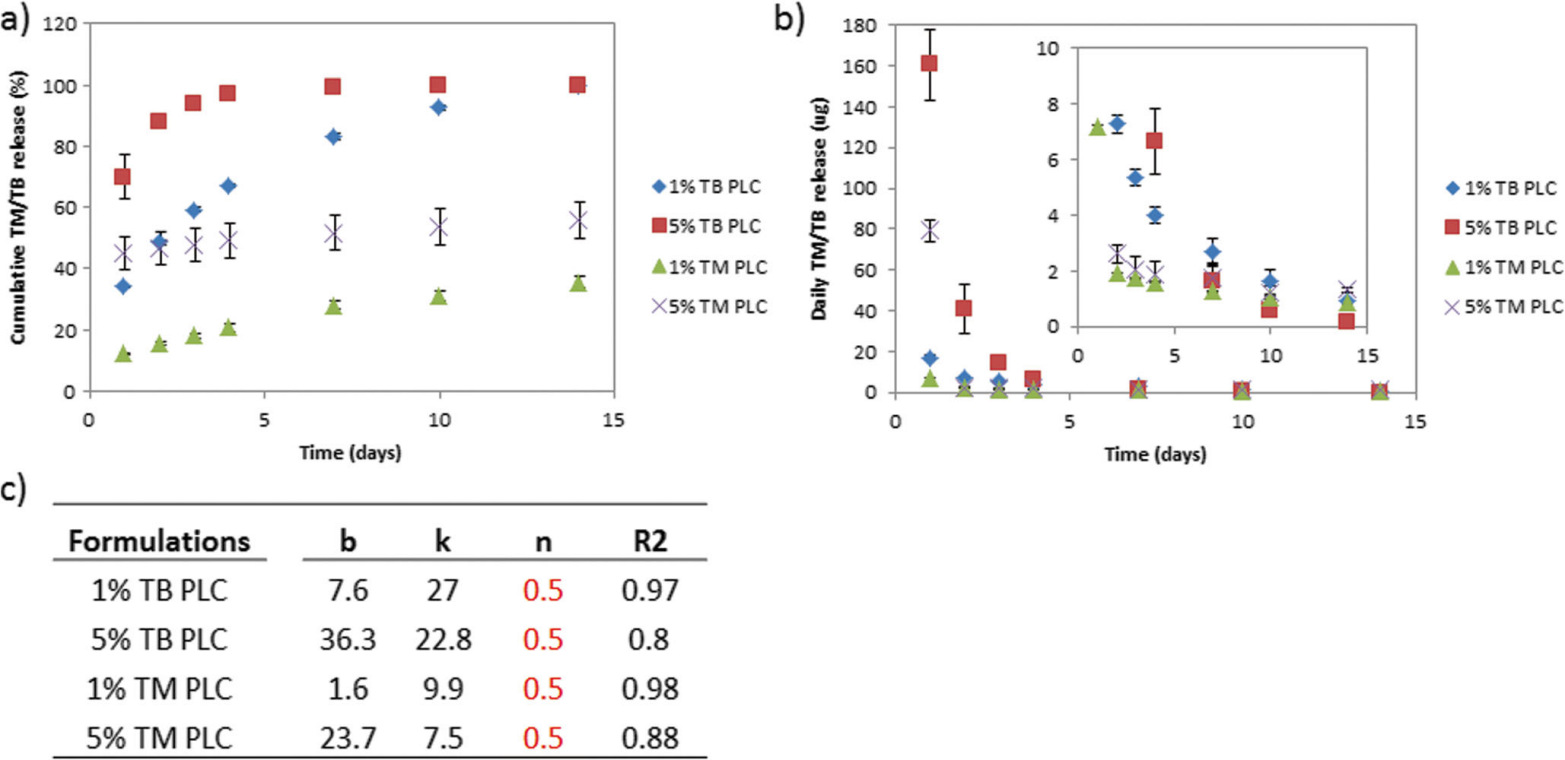
Release profile of 1 % TM, 1 % TB, 5 % TM, and 5 % TB PLC formulations. **a** Cumulative release of TM- and TB-loaded films. **b** Amount of TM/TB released each day. **c** Compilation of parameters upon fitting to the power law

Contrary to this expectation, Fig. 1a shows that the TB-loaded films showed faster overall release kinetics, including a substantially higher burst, compared to the TM-loaded films. The 1 and 5 % TB-loaded films released 34 and 70 % on day 1, and both displayed inopportune termination of release within 2 weeks and 1 week, respectively. On the other hand, the 1 and 5 % TM-loaded films showed more sustained release (beyond 2 weeks) compared to TB formulations due to lowered burst (Fig. 1b).

The reason for the higher burst amount (as designated by the magnitude of *b* in the fitting of the diffusion equation, in Fig. 1c) is that the TM is actually more lipophilic than TB. The reported value of the octanol/water partition coefficient for TB is 2.4 [19], while that for TM is about 60 [20]. This being the case, we used the more lipophilic TM for the rest of the studies.

#### Effects of blending

**Fig. 2:**
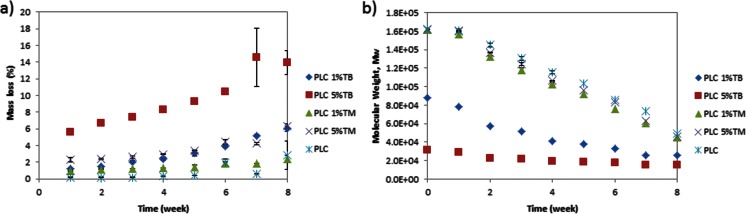
Percentage mass loss and molecular weight change of PLC neat film, 1 % TM, 1 % TB, 5 % TM, and 5 % TB PLC formulations. **a** Mass loss. **b** Molecular weight change over 8 weeks

Based on the above results, TM was selected as the drug form for optimization of release, and the matrix composition was further manipulated to get reduced burst and more linear release profiles. To modulate the burst release, PLC films were blended with PEG-based copolymers—PCL-PEG. This copolymer was chosen based on the hypothesis that the hydrophobic segments of the copolymer (PCL segments) could interact with the bulk matrix (PLC) and the hydrophilic segment of the copolymer could interact with TM to moderate the release (Fig. 2).

**Fig. 3:**
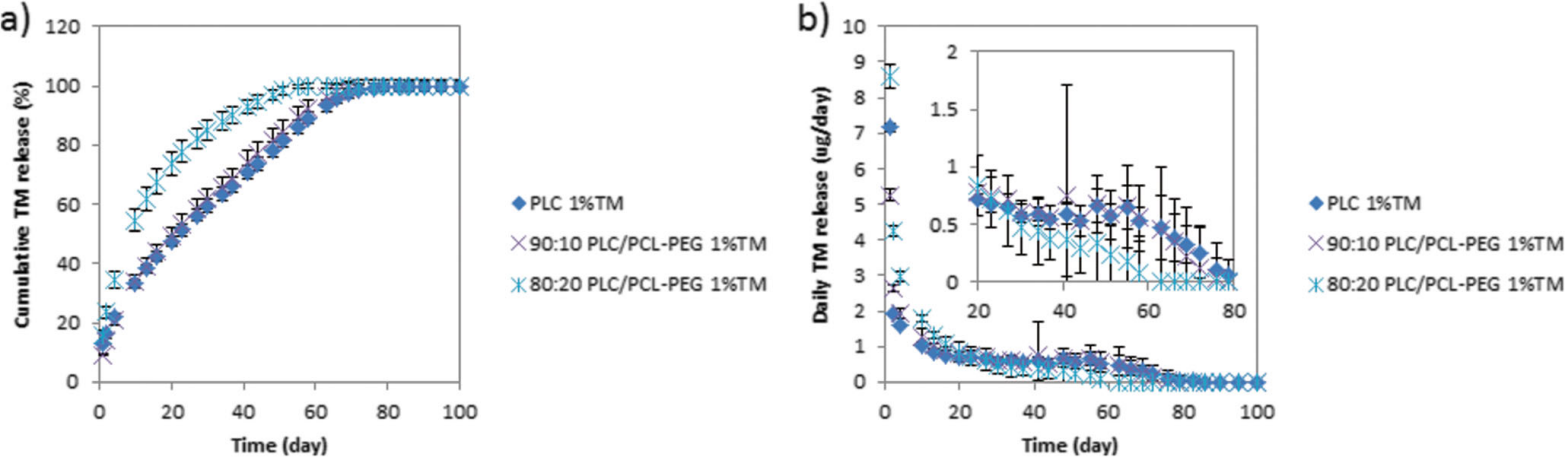
Release profiles of neat PLC, 80:20 PLC/PCL-PEG, and 90:10 PLC/PCL-PEG of 1 % TM-loaded formulations. **a** Cumulative release of TM. **b** Amount of TM released each day

Again, contrary to expectation, the release was actually higher for the blended than the neat film (Fig. 3a). Moreover, the burst release increased with increasing blending ratio (for example, the 10 % PCL-PEG blend formulation had a burst release of 9.3 % while the 20 % PCL-PEG blend had 15.8 % release on day 1). Following the initial release, the 20 % PCL-PEG blend showed average daily release amounts of 0.7 ± 0.6 μg for 76 days (Fig. 3b).

The fact that the blending of PLC with PCL-PEG did not effectively suppress the burst effect may be explained as follows: the idea behind the use of the PEG copolymer is to disperse the undissolved drug better in the matrix material, by interaction of the drug with the PEG segment and better anchoring through the presence of the attached PCL [21, 22]. At a PEG molar mass of 5000 Da, the segregation appears to be worse in that the blending forces more TM to the surface: this is either due to the higher-than-expected lipophilicity of TM and/or lesser interaction of the TM with the PEG component. As we will see in a subsequent section (“In vivo evaluation”), lowering the molar mass of the PEG block to 1000 Da results in better dispersion of the drug and leads to an almost linear profile, especially in a three-layer construct.

**Fig. 4:**
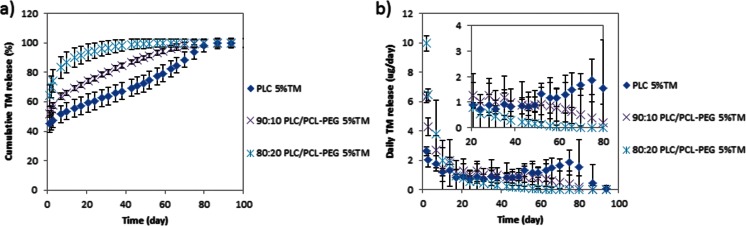
Release profile of neat PLC, 80:20 PLC/PCL-PEG, and 90:10 PLC/PCL-PEG of 5 % TM-loaded formulations. **a** Cumulative release of TM. **b** Amount of TM released each day

Since none of the 1 % loaded TM formulations meet the targeted dose of 2.5 μg/day, we investigated a higher drug loading of 5 % (Fig. 4a, b). Indeed, increasing the loading did increase the daily TM release, where 1.4 ± 1.4 μg/day of TM release was observed in the 20 % PCL-PEG blended formulation for 80 days. However, increasing the drug loading also greatly magnified the burst release, where drug release in the range of 90 ± 9.5 μg/day was observed on day 1 for all the 5 % TM-loaded formulations (Fig. 4b). The burst is possibly aggravated by the increased drug loading, which leads to phase-separated drug at the surface [23].

Based on the substantial burst effects seen at 5 % drug loading, it appears that a monolithic construct with the drug dispersed in a PLC matrix with or without the PEG copolymer would not be able to sustain release over a long period, which led us to pursue the use of “sandwich” constructs coupled with the use of a more selective compatibilizer in the following section.

#### Effects of a multilayered construct and polymer chain length

**Fig. 5:**
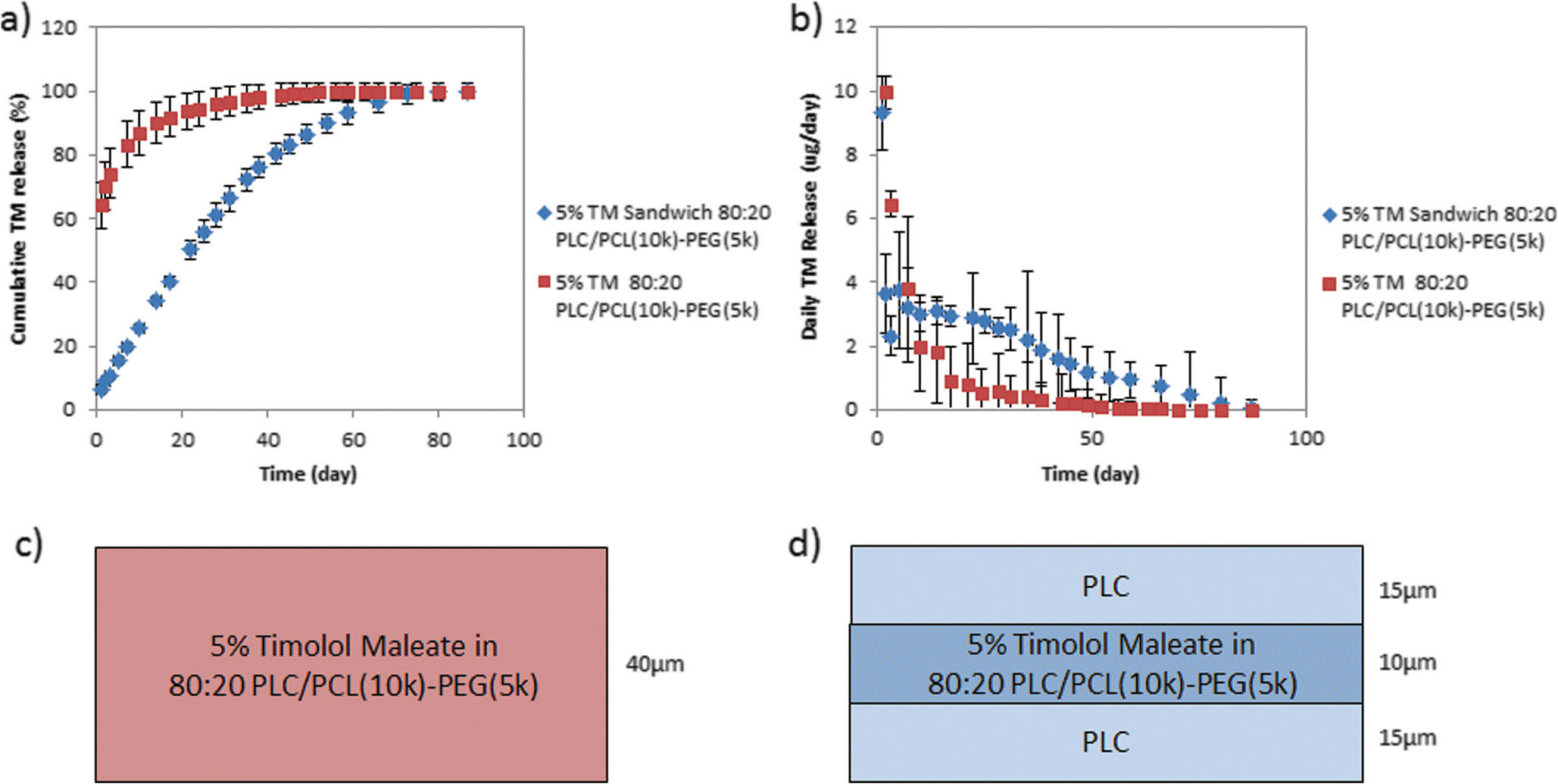
Release profile of homogenous 80:20 PLC/PLC-PEG and sandwich of PLC- 80:20 PLC/PCL(10k)-PEG(5k) -PCL 5 % TM-loaded formulations. **a** Cumulative release of TM. **b** Amount of TM released each day; schematic cartoon of **c** homogenous 80:20 PLC/PLC-PEG and **d** sandwich of PLC- 80:20 PLC/PCL(10k)-PEG(5k) -PCL 5 % TM-loaded formulations

To minimize the burst release, we investigated the use of drug-free barrier layers to sandwich the drug-loaded layer. As is evident from the results (Fig. 5), the introduction of the “sandwich” structure notably reduced the burst approximately ten times compared to the monolithic structure (Fig. 5a). It was also interesting that a zero-order release was observed, whereby the sandwich formulation released 2.1 ± 2.1 μg/day for 60 days following the initial release on day 1 (Fig. 5b). However, the introduction of the drug-free barrier layer also greatly limits the drug for loading, resulting in failure to achieve the desired target duration of 3 months.

**Fig. 6:**
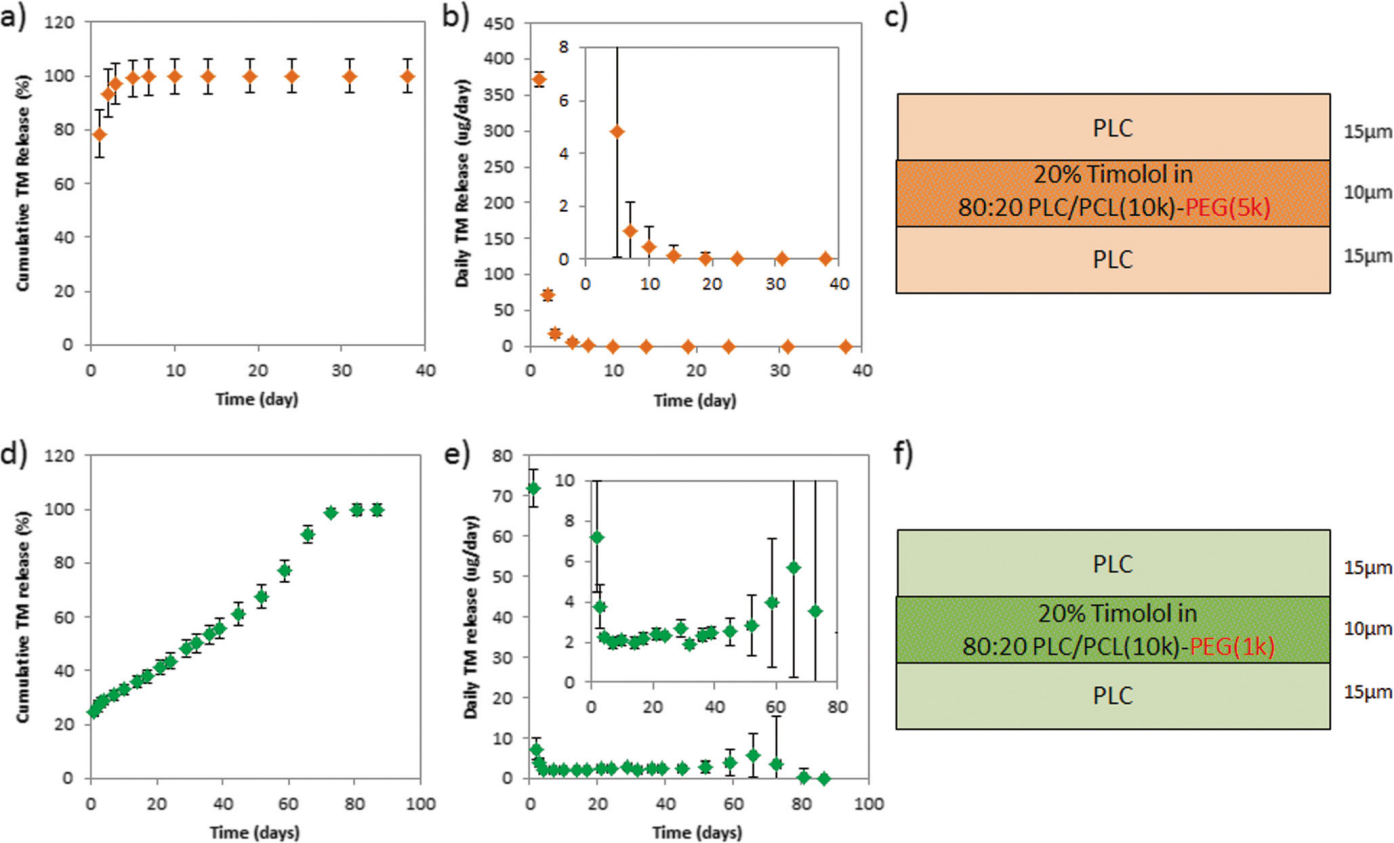
Release profile of sandwich 80:20 PLC/PLC-PEG (5 k) 20 % TM-loaded formulations. **a** Cumulative release of TM. **b** Amount of TM released each day. **c** Schematic of construct and release profile of sandwich 80:20 PLC/PLC-PEG (1 k) 20 % TM-loaded formulations. **d** Cumulative release of TM. **e** Amount of TM released each day. **f** Schematic of construct

Naturally, the increment in the thickness of the drug-loaded layer would extend the drug release period without drastically altering the release too much. But the subconjunctiva contains numerous small and fragile blood vessels [16–18] and increasing the dimensions of microfilms may potentially compromise patient safety, which is undesired. Hence, an increase in drug loading to 20 % was evaluated. Figure 6 shows the effects of polymer chain length in 20 % TM formulations blended using PCL-PEG with a 5-k PEG segment (Fig. 6a, b). The copolymer formulation with the 5-k PEG segment showed a burst release of 80 % on the first day. It appears that the barrier layers were not effective in limiting the burst release at the very high drug loading, due to the lack of anchorage of the drugs within the drug-loaded layer, and the resulting migration of the drug to the drug-free layers. Theoretically, the barrier layer could be strengthened by increasing the thickness and therefore increasing the diffusion distance for the drug to exit [24, 25]. However, for our work, it is impractical to increase the thickness of the barrier layer because the final thickness of the microfilm is only a mere 40 μm and the current barrier layers are already 15 μm in thickness each.

We believe that a lower molar mass of the PEG segment results in better drug dispersion and a more stable co-localization of the drug in the reservoir matrix. Indeed, in the formulation blended with the 1-k PEG segment copolymer, the burst release was effectively suppressed by the barrier layer (Fig. 6c). Furthermore, from this formulation, we achieved the desirable zero-order release with approximately 2.7 μg of daily TM release for 3 months (Fig. 6d). Indeed, the use of the copolymer with shorter PEG segment in the drug-loaded layer along in a sandwich construct was effective in retarding the burst and modulating the release. As alluded to earlier, the effective suppression of burst by the drug-free layer in the 1-k PEG segment formulation can be attributed to this factor: the lower PEG molar mass may also allow for better dispersion of the PEG copolymer phase within the PLC matrix, due to a lesser mismatch between the copolymer and matrix. It should be noted that the release profiles remain unchanged after storage for 4 months at ambient conditions, in a dessicator.

This formulation (sandwich 80:20 PLC/PLC-PEG (1 k) 20 % TM-loaded formulations) gave an excellent zero-order release while attaining the daily therapeutic dose over several days. Since this formulation more than adequately matches our requirements, it was selected for further in vivo evaluation.

### In vivo evaluation

**Fig. 7:**
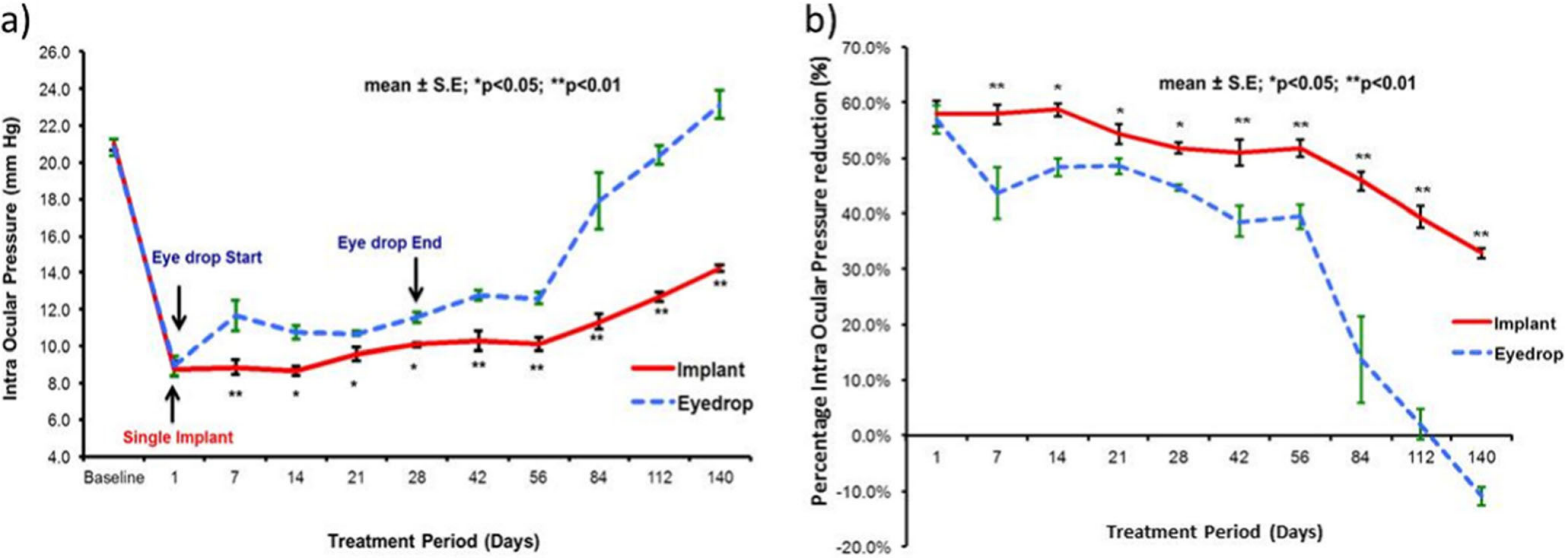
Comparison between groups with TM eyedrop treatment and TM implant treatment. **a** Intraocular pressure (IOP) and **b** percentage reduction of IOP

To confirm whether the extended duration of release translates to longer efficacy of action, we evaluated the safety and efficacy of TM-loaded microfilms in ocular hypersensitive non-human primates [26]. Since IOP is the key modifiable risk factor in glaucoma, the IOP reduction following subconjunctival injection of TM-loaded microfilm was compared to the IOP reduction achievable with daily topical administration of TM (eyedrops). Topical treatment using 0.25 % of TM for 28 days showed effective reduction of IOP during the treatment period (Fig. 7a). The topical treatment used in this work matches the current regimen used by glaucoma patients [27, 28]; hence, effective IOP reduction was expected. However, after cessation of treatment, the IOP returned to the hypertensive baseline IOP after a few weeks following a washout effect from the eyedrop medication. In the TM microfilm group, the IOP was suppressed for the entire duration of the experimental period of 140 days (Fig. 7a).

**Fig. 8:**
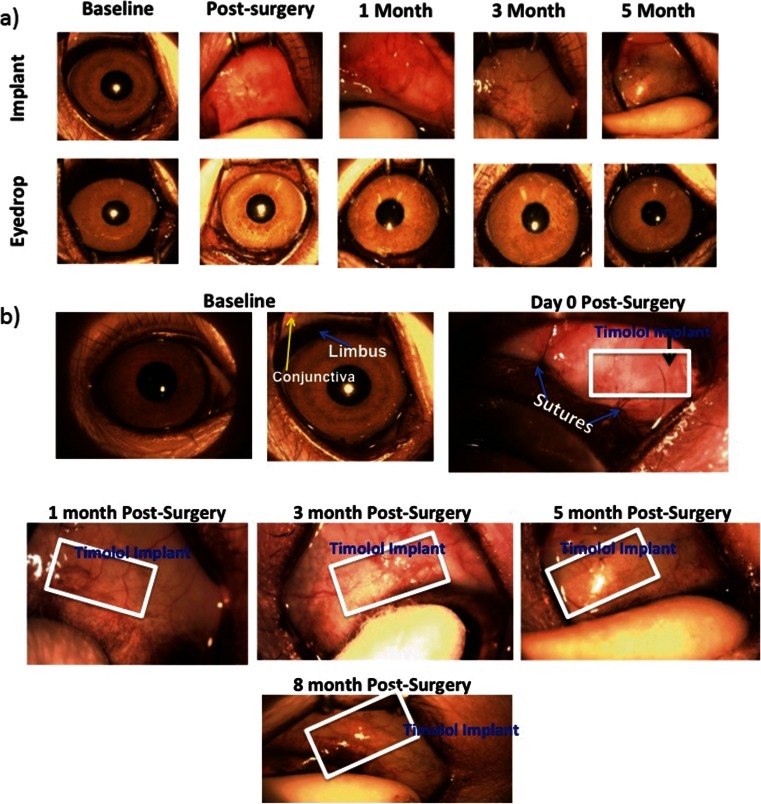
**a** Slit lamp photographs of subconjunctivally implanted TM microfilms and the eyedrop treatment group at 1 month, 3 months, and 5 months after treatment. **b** Implant was noted at 0 day, 1 month, 3 months, 5 months, and 8 months after insertion

To evaluate the IOP-lowering effects, the percentage reduction of IOP is presented in Fig. 8b. The daily TM dosing showed an IOP decrease of 45.8 ± 6.3 % between day 1 and 56; subsequently, IOP reduction plummeted to 1.6 ± 12.3 % between day 56 and 140 (Fig. 8b). In the TM microfilm group, percentage IOP reduction was 50.1 ± 8.5 % throughout 140 days of the experiment. Further analysis was performed to compare the IOP reduction at each time point, and the TM microfilm group displayed significant IOP reduction. The changes in IOP between the topical administration and TM microfilm were compared, and the analysis showed statistical significance over the treatment time (*P* < 0.005), suggesting that the TM effectively reduces the IOP compared to the topical administration (*P* < 0.005). In addition, from the slit lamp examinations in Fig. 8a, b, there is no evidence of protrusion or dislocation of the microfilm in the eyes. There were also no signs of infection, neovascularization, or bleeding at the sites of insertion. In this work, we did not observe any TM side effects in the monkeys used in this study. However, it is important to note that systemic adsorption of TM has also been reported to cause respiratory and cardiovascular side effects [29, 30] including nocturnal hypotension, bradyrhythmias, and bronchospasm in patients. Other ocular-related side effects such as unexplained deep orbital pain, blurred vision [31], and eye redness in patients [32] have also been reported.

The encouraging in vivo results indicate that TM microfilms offer a significantly improved treatment option in glaucoma management. It should be noted that the in vivo effects (5 months) lasted longer than the in vitro release (approximately 3 months). There is a possible explanation for longer in vivo effects (5 months) when compared to in vitro release (approximately 3 months). It is likely that the clearance from the subconjunctival space is slower than the in vitro “clearance” (i.e., daily replenishment of release medium). Nevertheless, it is evident that we have developed a TM sustained-release microfilm that is safe and effective in lowering IOP for 5 months in vivo.

## Conclusion

By using a combination of multilayering and blending with PEG copolymers, we were able to develop a timolol maleate-incorporated biodegradable film that can deliver TM at a therapeutic dose for 90 days in vitro. In primates with ocular hypertension, the film provided sustained IOP lowering for up to 150 days. This study therefore presents a TM microfilm that can provide a significantly different therapeutic option for glaucoma management, surmounting the challenges of patient non-adherence to the cumbersome topical regimen.
